# Mortality in Miners with Coal-Workers’ Pneumoconiosis in the Czech Republic in the Period 1992–2013

**DOI:** 10.3390/ijerph14030269

**Published:** 2017-03-07

**Authors:** Hana Tomášková, Anna Šplíchalová, Hana Šlachtová, Pavel Urban, Zdeňka Hajduková, Irena Landecká, Rostislav Gromnica, Petr Brhel, Daniela Pelclová, Zdeněk Jirák

**Affiliations:** 1 Institute of Public Health, Ostrava 70200, Czech Republic; anna.splichalova@zuova.cz (A.S.); hana.slachtova.x@gmail.com (H.S.); zdenek.jirak@osu.cz (Z.J.); 2 Department of Epidemiology and Public Health, Faculty of Medicine, University of Ostrava, Ostrava 70103, Czech Republic; 3 The National Institute of Public Health, Prague 10042, Czech Republic; pavel.urban@szu.cz; 4 Department of Occupational Medicine, 1st Faculty of Medicine, Charles University in Prague, Prague 12108, Czech Republic; daniela.pelclova@LF1.cuni.cz; 5 Clinic of Occupational Health and Preventive Medicine, University Hospital in Ostrava, Ostrava 70852, Czech Republic; zdenka.hajdukova@fno.cz; 6 Centre of Occupational Medicine, Miners’ Hospital in Karvina, Karvina 73506, Czech Republic; irena.landecka@seznam.cz; 7 Department of Occupational Medicine, Miners’ Clinic, Ostrava 70200, Czech Republic; gromnica@lazneluhacovice.cz; 8 Department of Occupational Medicine, St. Anne’s University Hospital and Faculty of Medicine, Masaryk University in Brno, Brno 62500, Czech Republic; petr.brhel@fnusa.cz; 9 Department of Physiology and Pathophysiology, Faculty of Medicine, University of Ostrava, Ostrava 70103, Czech Republic

**Keywords:** lung cancer, chronic obstructive pulmonary diseases, coal dust, silica

## Abstract

While working underground, miners are exposed to a number of risk factors that have a negative impact on their health and may be a cause of an increased mortality in miners. The aim of the study was to compare total and specific mortality in black coal miners with acknowledged coal-workers’ pneumoconiosis (CWP) and without CWP, and the mortality of the general male population in the Czech Republic in the period 1992–2013. The sample consisted of 3476 coal miners with CWP and 6687 ex-coal miners without CWP, who were removed after achieving the maximum permissible exposure (MPE). The mortality risk differences were analyzed with the use of the standardized mortality ratio (SMR) and 95% confidence interval. Significantly higher total mortality (SMR = 1.10; 95% CI: 1.02–1.17), and mortality from malignant neoplasm (SMR = 1.16; 95% CI: 1.03–1.30), lung cancers (SMR = 1.70; 95% CI: 1.41–2.04), and non-malignant respiratory diseases (SMR = 2.78; 95% CI: 2.32–3.31) were found in the sample of coal miners with CWP. In this sample, the severity of CWP was assessed, and the SMR increased with the severity of CWP. The total (SMR = 0.86; 95% CI: 0.82–0.91) and specific mortality of miners without CWP were not higher compared with the general population. In the case where the miners were removed from underground work after achieving the MPE (without CWP), their mortality was not higher than that of the general population, but the mortality of miners with CWP was higher compared to the general population. This mortality was affected by malignant and non-malignant respiratory diseases.

## 1. Introduction

Black coal miners (further referred to as only coal miners) are exposed to a number of risk factors at work. For physical factors, they are exposed to noise, vibrations transmitted to the hands, a high working and heat load associated with the loss of up to 4 L of water/per work shift through sweat and breathing, and an overloading of the upper extremities when working with pneumatic tools. The most serious factor that significantly threatens the health and life of miners, however, is mine dust containing crystalline forms of silica (SiO_2_). Compared with the general population, the adverse effect of occupational exposure and conditions will appear later in time for this profession.

Czech underground coal mining is a job with a high risk of coal-workers´ pneumoconiosis (CWP), rapidly progressing even after removal from the mine from mild to severe forms of massive pneumoconiosis, often leading to death before reaching 50 years of age. CWP is an occupational disease conditioned by long-term exposure to coal dust containing up to 15% crystalline forms of silica (SiO_2_). In the early stages of the disease, nodules with a mild inflammatory reaction conditioned by centriacinar accumulation of macrophages containing coal dust are formed. Long-term exposure leads to the formation of large nodes that significantly reduce lung function and consequently may lead to heart failure. CWP was recognized as an occupational disease of miners in the Czech Republic (CR) [1].

In the CR, a number of studies were conducted that resulted in the determination of preventive measures. Technical measures to reduce dust levels were established in the CR in the 1960s. The effectiveness of these measures was monitored by regular measurement of the total and respirable fractions at all mining sites, including the monitoring of silica content in the respirable fraction of dust. The average, time-weighted concentrations of respirable fractions of dust in the period 1962–1989 amounted to 4.33 mg/m^3^ for coal extraction workers, 2.20 mg/m^3^ for drifters, and the average concentrations of quartz in the respirable fraction of dust reached 1.9% ± 0.8% and 3.8% ± 2.2%, respectively. Based on the analyses of the measured results of concentrations of the respirable dust fraction, the content of quartz, radiographs of the lungs, and the specified content of deposited dust and silica in the lungs of deceased miners, the maximum permissible exposure (MPE) of dust was determined. The MPE was determined for specific mining sites and professions (drifters, coal extraction workers, and others) on the level of probability that 5% of miners will become ill with a mild form of CWP after achieving the MPE. According to the study from 1978 [2], the average permissible cumulative dose of the respirable fraction of dust was 154,464 mg for coal extraction workers (76,840 mg for drifters) and the corresponding cumulative dose of quartz of 2925 mg for coal extraction workers (2934 mg for drifters resp.) is consistent with the determined MPE values for professional coal extraction workers/drifters. The MPE verification was carried out in 1993 and 1997 [3,4] and, subsequently, at regular intervals. Based on this follow-up, it was found that less than 5% of miners became ill with a mild form of CWP in compliance with the MPE. The preventive replacement of miners after achieving the MPE was first introduced in the largest mining region—the Ostrava-Karvina mines (OKM)—and in 1991 it was implemented in the entire country. In the same year, the National Registry of Occupational Diseases was established, which has enabled monitoring of all CWP cases for the whole CR.

Published reports mostly indicate both significantly higher total mortality and specific mortality from non-malignant respiratory system diseases in miners [5,6,7,8,9,10,11,12,13,14]. In relation to exposure to dust containing SiO_2_, epidemiological studies have been focused mostly on the issue of the risk of malignant neoplasms of the lungs and stomach in recent years. Regarding the mortality of miners from malignant neoplasms of the lung, the results of the individual studies vary from negative associations [6,7,13,15,16], to not significantly increased risks, to a statistically significant increase of values [10,14,16,17,18,19]. In some cases, large cohorts are followed up for a period of several decades, e.g., the US study of underground coal miners [5,6], where a recent study was in compliance with the previous studies that had found higher mortality from malignant and non-malignant respiratory diseases [20]. Numerous authors explored the relationship between the exposure to dust in coal mines and mortality from malignant neoplasms of the stomach and the digestive system, respectively. The increased mortality risk of stomach cancer was reported by most of the authors, but the increase was not statistically significant [5,9,10,12,21]. The study of Miller et al. investigated another large cohort consisting of British coal miners [9,10].

In our previous study [22] which dealt with the analysis of cancer risk in miners in the period 1992–2001, the statistically significant risk of incidence of malignant lung neoplasms in the miners with CWP was confirmed, but not in miners without CWP and the initial form of CWP. The level of risk increased with the severity of CWP.

The aim of the present study is to compare the total and specific mortality in coal miners with CWP and in ex-coal miners (the ones that were removed due to the achieved MPE) without CWP, and the mortality of the male population of the CR in the period 1992–2013. The total and specific mortality in coal miners with CWP were analyzed according to the severity of CWP.

## 2. Materials and Methods

### 2.1. Study Design and Data

The first cohort (coal miners with CWP) consisted of 3476 former coal miners who were recorded in the National Registry of Occupational Diseases because of CWP diagnosis in the years 1992–2013 from all mines in the CR where the black coke coal and energy coal is mined. From the National Registry of Occupational Diseases, the date in the reports on occupational diseases contained, namely: the date of birth, the name of the workplace, the length of exposure in years and the classification of CWP according to the International Labor Organization (ILO) (1980) [23]. Four forms of CWP were distinguished from initial forms of CWP to CWP in association with tuberculosis—the initial form of CWP (iCWP) (size of rounded opacities p2, q1, r1), the simple CWP (sCWP) (size of rounded opacities p3, q2, r2, q3, r3), progressive massive fibrosis (PMF) (A, B, C), and CWP in association with active tuberculosis (CWP & TBC) (all sizes of rounded opacities including A, B, and C). In the case of iCWP the condition for acknowledgement of occupational disease is age up to 40 years, and exposure up to 15 years (3000 shifts) [1]. The data on smoking (smoker, non-smoker, ex-smoker) were completed by workers from the reporting centers of the medical reports from the relevant departments (clinics) that had diagnosed the disease.

The second cohort was created from the OKM miners (N = 6687), who were removed due to the achieved MPE in the years 1986–1999. These miners did not become ill with CWP until the year 2013 and in the beginning of the follow-up (1992) they were alive. This cohort had already been analyzed in 2001 [22]. For these miners, recorded information included: the date of birth, mining profession, length of exposure in years, percent of the MPE, the year of ceasing work in mine, and smoking status.

All persons in the both cohorts were men. The personal and professional data of miners was linked to the data in the National Population Registry, where the information on the date and diagnosis of death (according to the International Classification of Diseases revision 9 (ICD-9) and 10 (ICD-10)) was obtained for the period from 1992 to 2013.

Total mortality (diagnosis (dg.) 001–999 according to the ICD-9/A00–Y98 according to the ICD-10) and specific total cancer mortality (dg. 140–239/C00–C97), malignant neoplasms of the respiratory system (dg. 162/C33–C34 further lung cancers), diseases of the circulatory system (dg. 390–459/I00–I99), non-malignant respiratory diseases (dg. 460–519/J00–J99 further NMRDs), chronic obstructive pulmonary disease (dg. 496/J44 further COPDs), and coal-workers´ pneumoconiosis (dg. 500/J60) were followed in miners. Mortality was monitored in both cohorts and also in individual subgroups according to the severity of CWP.

### 2.2. Statistical Analyses

The risk of death in coal miners compared with the population of males in the Czech Republic (further reffered to as the general population) was assessed by calculating the standardized mortality ratio (SMR) [24] and 95% confidence interval (CI). This analysis was based on data on total mortality and specific mortalities by five-year age groups (30–85 and over 85 years) for the period 1992–2013. Expected deaths were calculated by applying the age-specific and calendar year-specific mortality rates for all Czech men.

Information about the age-specific mortality of men in the CR was based on data from the National Health Information System for the period 1992–2013. The analysis of variance (ANOVA), the Bonferroni test, and χ^2^ test were used for statistical analyses. Statistical tests were evaluated at a significance level of 5%. Data analyses were performed using Stata version 14 (StataCorp LP, College Station, TX, USA) [25].

### 2.3. Ethical Consideration

The study was approved by the Ethics Committee of the Institute of Public Health in Ostrava, Czech Republic, reference number 1/2014.

## 3. Results

### 3.1. Description of the Cohort of Coal Miners with CWP

The cohort of coal miners with CWP (N = 3476) consisted of coal miners from the five mining sites in the Czech Republic (the OKM, the East Bohemia coal mines, the West Bohemia coal mines, the Rosice coal mines, and the Kladno coal mines). The highest proportion (80%) was composed of miners from the OKM.

**Figure 1:**
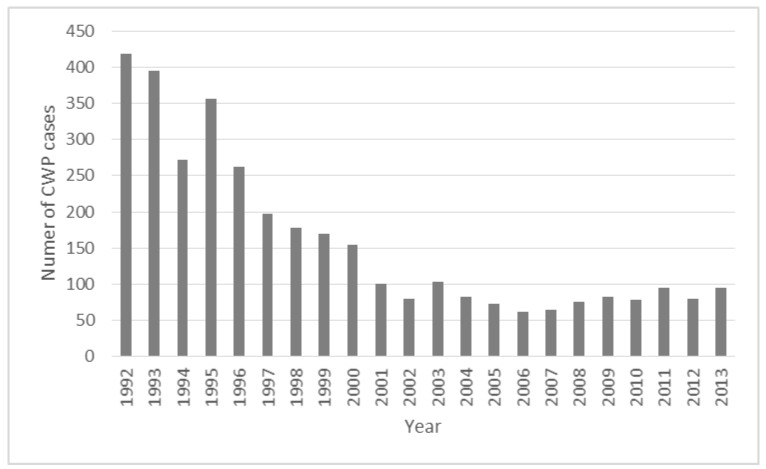
The number of newly reported cases of CWP in individual years.

The annual number of newly reported cases with the compensated form of CWP decreased during the reporting period from 418 in 1992 to the range of 62–104 cases in the years 2001–2013 (Figure 1).

The average age of the coal miners was 49.7 ± 12.4 (mean ± standard deviation) years at the time of recognition of occupational disease and the mean duration of exposure in the mining profession was 20.7 ± 7.8 years. The data on exposure was obtained in 94.5% of the sample. Data on smoking was detected in 77.6% of the sample. The proportion of smokers and ex-smokers in the cohort of coal miners with CWP, for whom the data on smoking status was available, reached 66.6% (21.8% ex-smokers and 44.8% smokers).

In coal miners with CWP, four forms of CWP were distinguished, from the initial form of CWP to CWP in association with tuberculosis. The group of individuals with the simple CWP accounted for the largest proportion. According to the forms of CWP, statistically significant differences were found in the age of CWP acknowledgement, the duration of exposure, and the proportion of smokers (Table 1). The highest age and duration of exposure were found in coal miners with PMF, and the most smokers and ex-smokers were identified in coal miners with CWP & TBC.

In total, 889 persons died in the reporting period 1992–2013, which is 25.6% of the coal miners with CWP. The mean age at death was 67.4 ± 11.84 years. The most common cause of death was disease of the circulatory system and malignant neoplasms (MN), shown in Table 2. Out of the malignant neoplasms, lung cancers were the most frequent, accounting for 13.0% of all deaths. Non-malignant diseases of the respiratory system were the third leading cause of death, out of which CWP was listed as the cause of death in 4.2%, and COPDs in 5.2% of the deceased miners.

The coal miners with CWP, whose cause of death was digestive system diseases, or injuries, poisoning, and certain other consequences of external causes, died at a significantly younger age (*p* < 0.001) compared to those who died of other causes (Table 2); similarly, it was with respect to the length of exposure.

### 3.2. Description of the Cohort of Coal Miners without CWP and the Comparison of Both Cohorts

A basic description of the cohort is shown in the Table 1. The cohort of coal miners without CWP (N = 6687) was created by the miners from the OKM, and the median value of the percentage of MPE was 100% (interquartile range 98%–133%). The average age of miners without CWP when entering the study (age in 1992 or age at time of cessation of work underground when leaving after 1992) (44.0 ± 6.3 years) was significantly lower (*p* < 0.001) than in coal miners with CWP, namely by an average of 5.7 years. The duration of exposure was significantly longer (*p* < 0.001). The information on professional exposure was detected in all miners with CWP and information on smoking was obtained in 99.8% of coal miners. The proportion of smokers and ex-smokers (67.3%) was comparable with this proportion in the cohort of coal miners with CWP (*p* = 0.508), but in the cohort of coal miners without CWP, the proportion of smokers was higher (58.8%), while the percentage of ex-smokers was lower (15.5%) (*p* < 0.001).

In total, 1320 deaths (19.7%) were found in the cohort of coal miners without CWP during the period 1992–2013. The average age at death was 59.9 ± 8.0 years, and the age of death was correlated with the age at study entry (r = 0.71). Similarly, as in coal miners with CWP, the diseases of the circulatory system and MN were the most common cause of death (see Table 2). Lung cancer was the reported cause of death in 11% of deceased persons (see Table 3). The third most frequent cause of death in coal miners without CWP, unlike the coal miners with CWP, was injury, poisoning, and certain other consequences of external causes (S00-T98), which accounted for 10% of deaths. In these deaths, the lowest age at death was detected (*p* < 0.001). Diseases of the respiratory system represented 6% of deaths. In 14 (1.1%) miners, CWP was reported as the cause of death. The proportion of smokers and ex-smokers in deceased persons between the cohorts differed significantly (*p* < 0.001); in the cohort with CWP, the proportion of smokers was 45.5% and ex-smokers 23.4%; in the cohort without CWP, the proportion of smokers was higher (65%) and the proportion of ex-smokers was lower, accounting for 14.6% (Table 3).

### 3.3. Total and Specific Mortality—Both Cohorts

A higher statistically significant total mortality was found in the coal miners with CWP compared with the general population (Table 3). The increased mortality of miners was caused, primarily, by an increased specific mortality for lung cancers (SMR = 1.70) and NMRDs (SMR = 2.78). The contribution of COPDs significantly increased the mortality for NMRDs; the mortality rate was more than three times higher among the coal miners with CWP than in the general population. Conversely, the risk of death for cardiovascular diseases was lower among the coal miners with CWP (Table 3).

In the coal miners without CWP, total mortality, mortality from MN, lung cancers, and diseases of the circulatory system were significantly lower compared with the general population. The detected differences between the deaths from NMRDs, COPDs, and diseases of the digestive system were not statistically significant (Table 3).

### 3.4. Total and Specific Mortality According the Severity of CWP

From Table 4, it is apparent that the total and specific mortality of miners with CWP was comparable to the mortality of the general population. In the group of miners with sCWP, the total mortality rate was also comparable with the total mortality of the general population; however, the specific mortality for lung cancers and NMRDs was significantly higher. In the groups of miners with PMF and CWP & TBC, the total and specific mortality for malignant neoplasms, lung cancers, and especially NMRDs were significantly higher.

COPDs as the cause of death was found in 46 cases. In the group of miners with iCWP, COPDs was diagnosed as the cause of death only in two cases, and mortality was higher compared with the general population, but not at a statistically significant level. In other CWP categories, the mortality for COPDs was significantly higher (Table 4). The severity of CWP was significantly associated with the average age at time of death (p < 0.001) and the mean duration of exposure (p = 0.013) of the deceased miners (Table 4). A strong correlation was found between age at reporting and age at death (r = 0.88). On the contrary, a weak correlation was identified between the age at death and the length of exposure (r = 0.35). The data on exposure was missing in 14.6% of deceased miners. Data on smoking was available for 67.7% of deceased miners. A statistically significant difference was found between the proportion of non-smokers and smokers (including ex-smokers) in the specific groups according to the severity of CWP (p = 0.015). The highest prevalence of smokers was identified in the category CWP & TBC and the lowest in the category PMF.

## 4. Discussion

### 4.1. Mortality in Both Cohorts

The comparison of mortality in the cohorts of miners with and without CWP has enabled the evaluation of the effectiveness of the MPE in relation to the mortality of black coal miners. The SMR of both cohorts was calculated based on a comparison with the general male population in the CR. The total mortality in miners with CWP was significantly higher (SMR = 1.10, 95% CI: 1.02–1.17) compared with the general population in the CR. Increased specific mortality for lung cancer and NMRDs contributed to the increased total mortality. The total mortality in miners without CWP was significantly lower than in the general population (SMR = 0.86, 95% CI: 0.82–0.91); no increase of any specific mortality was found in this cohort compared with the general population.

Based on the comparison of the mortality of miners with CWP (including all forms of CWP severity) and the mortality of miners without CWP with the general population, we can assume that the preventive measure MPE is effective also in relation to mortality. The measure of the MPE prevents CWP or allows only a mild form of the disease and, thus, reduces the mortality of coal miners to the level of the general population.

### 4.2. Mortality According to the Severity of CWP

In the sample of miners with CWP according to severity, the category sCWP (71%) was the most frequent, followed by iCWP (17%), PMF (7%), and CWP & TBC (5%). The iCWP group was created by susceptible persons who became ill with CWP at a young age (around 40 years of age) and after a short exposure (<15 years). A higher mortality, compared with the general population in the CR, in these persons was not found in the current study, so the question that exists is whether the relationship will change with the aging of the cohort. In the sCWP group, total mortality was comparable to the general population, but mortality from lung cancer and NMRDs was significantly higher. The increased mortality was affected primarily by CWP itself, and also by COPDs. On the contrary, a significantly lower mortality from the diseases of the circulatory system was found, which can be explained by the earlier deaths from NMRDs or lung cancer. The PMF group predominantly includes individuals for whom the replacement criteria have not already been applied; hence, the total dust exposure was higher than determined according to the MPE. This group is relatively less represented (N = 233), which may affect the accuracy of the results. Out of this group, 41% of persons died, and the total mortality was higher compared with the general population, although this increase was not statistically significant. The mortality for lung cancer and NMRDs was, again, significantly higher than in the general population. In the group of miners with CWP & TBC, TBC also probably contributed to the statistically higher total mortality that was contributed mainly by NMRDs and lung cancer.

### 4.3. Comparison with Other Studies

The mortality of black coal miners with CWP was studied by Starzyński et al. [13] and Meijers et al. [8]. A significantly increased total mortality (SMR = 1.05; 95% CI: 1.00–1.10) was found in Polish coal miners with CWP [13]. In Dutch miners with CWP [8], a significantly higher total mortality compared to the general population was identified (SMR = 1.27, *p* < 0.05). In other studies, the mortality of miners, regardless of the presence of CWP, was analyzed. Some of these studies investigated the cohort of U.S. coal miners [5,6,20] in which an increased mortality was not found. Another large study analyzed the cohort of British miners [7,9,10] in which the total mortality was higher compared to the general population, but the increase was not statistically significant (SMR = 1.009; 95% CI: 0.990–1.029). Similar results were found in other studies [12,14].

Epidemiological studies show that CWP and COPDs represent the main contributions to the increased mortality rate for NMRDs in miners, and the risk of both diseases increases with the duration of exposure and the cumulative dose of dust [8,10,11,26].

The risk of death for NMRDs in the analyzed cohort of miners with CWP increased with the severity of CWP, ranging from simple CWP to more than triple in the cases of complicated CWP and CWP in association with active TBC. While in the case of CWP a professional etiology is undisputed, the COPDs have been developing even among people outside the exposure to dust. Boschetto et al. [27] considered that about 15% of COPDs arise in relation to dust exposure. In the study sample, the mortality for COPDs was approximately three times higher compared to the general population. Despite the relatively low numbers, the risk of death for this diagnosis significantly rose according to the severity of CWP.

### 4.4. Lung Cancer

Whereas the increased risk of death for NMRDs in coal miners has been indicated by most authors, opinions on the risk of lung and stomach cancers vary. Stomach cancer was stated as a cause of death in 13 persons in the present study of miners with CWP. Our previous study that focused on cancer incidence did not find a statistically significant difference compared to the general population [22], which was the reason to exclude this diagnosis from the current analysis. Most authors found the risk of dying from lung cancer comparable to or only slightly increased from the population [6,7,13,15]. A statistically significant risk of death from lung cancer (SMR = 1.57) was indicated by Morfeld et al. [19] for German coal miners. This finding was attributed to the high quartz content (8%) in the coal dust. Latz et al. [17] concluded, based on the results of a mortality study in German miners, that the risk of lung cancer in miners from the German mines cannot be clearly confirmed or denied. The work of Miller [10,20] and Japanese authors [14,17] highlighted a possible relationship between long-term exposure to dust in coal mines and the risk of lung cancer. In the present study, a statistically significant increased risk of lung cancer (SMR = 1.70) was found in the cohort of coal miners with CWP.

After splitting the sample into groups of miners according to the severity of CWP, an increased risk of death from lung cancer in all forms of CWP, starting with simple CWP, was found. In the group of miners with the initial form of CWP, and in the cohort of coal miners without CWP, the risk of dying from lung cancer was comparable to or lower than that of the general population. The influence of molds and ionizing radiation can be eliminated from other factors that could affect the increased incidence of lung cancer in the analyzed sample. Based on the study by Dobias et al. [28], it is possible to say that molds were not a risk factor in the Czech mines. Radon levels had been repeatedly measured by state control authorities (Regional Public Health Authority) and in the black coal mines no risk of radon was identified. In addition to working in the mine, TBC also probably contributed to a higher risk of lung cancer in the group of miners with CWP & TBC [29,30,31]. Smoking is another important risk factor of lung cancer and NMRDs [32].

In the Czech male population, non-smokers comprised 41.1% and smokers 59.9% (current smokers 32.4%, ex-smokers 26.5%) [33]. The prevalence of smokers in the cohorts of miners was higher compared with men in the CR. The proportion of smokers and ex-smokers in both cohorts was comparable, i.e., 66.6% in the cohort of coal miners with CWP and 67.3% in the cohort of coal miners without CWP. The reason for determining the proportion of smokers and ex-smokers was that information on smoking in miners with CWP was collected from the medical records and it was not known to what period the information was related. In miners without CWP, information was obtained at the time of replacement from the mine. The different recording times of detection of the data on smoking could explain the difference in the proportion of ex-smokers being 21.8% of the cohort of coal miners with CWP and 13.5% of the cohort of coal miners without CWP.

Missing data on smoking in 27% of coal miners with CWP could represent a limitation of the study. However, the percentage of smokers (including ex-smokers) was the lowest in the group with PMF and the risk of malignant and non-malignant neoplasms of the respiratory system in this group was significantly higher. Based on the same proportion of smokers in the cohorts, and the above findings, we can consider that smoking should not present a confounding factor in this study.

We consider that the increased mortality in miners with CWP is conditioned by an increased production of reactive oxygen species (ROS) due to the inhalation of dust containing SiO_2_. It is assumed that the ROS led to damage of the nuclear and mitochondrial DNA and cellular proteins and, thus, accelerated the aging process of the organism [34].

## 5. Conclusions

A significantly higher total mortality and mortality from malignant and non-malignant respiratory diseases was found in the miners with acknowledged CWP. Mortality for non-malignant respiratory diseases was mostly influenced and affected by CWP and chronic obstructive pulmonary disease. In the group of coal miners with the initial form of CWP, the total and specific mortality were comparable to the mortality of the general male population in the Czech Republic. In other groups, the SMR values increased with the severity of CWP.

Miners without CWP, who were removed from the underground workplace after they achieved MPE, had a significantly lower total mortality, mortality due to malignant neoplasms, and diseases of the circulatory system compared with the general population, which probably reflects a “healthy worker effect”. Mortality for NMRDs and diseases of the digestive system was comparable with the population data.

The analysis of data on miners with and without CWP will continue and will be focused on the incidence of cancers and the analysis of available risk factors. A possible bias of cancer risk will be analyzed which might be caused in cases where cancer mortality, but not cancer incidence, is followed up.

## Figures and Tables

**Table 1:** Description of the cohorts of coal miners without and with coal-workers´ pneumoconiosis (CWP) and according to the severity of CWP.

Category of CWP	Number	%	Age at Study Entry ^1^ (Year)		Length of Exposure (Year)		Proportion of Smokers ^2^
			Mean	SD	Mean	SD	%
Without CWP	6687	100%	44.0	6.3	22.9	5.9	67.3%
With CWP	3476	100%	49.7	12.39	20.7	7.80	66.6%
iCWP	606	17%	40.4	6.92	18.1	6.21	65.3%
sCWP	2465	71%	50.9	11.88	21.3	7.86	66.7%
PMF	233	7%	59.9	13.58	22.5	9.00	62.4%
CWP & TBC	172	5%	50.2	14.13	18.8	8.20	77.0%
*p*-value ^3^			*p* < 0.001		*p* < 0.001		*p* = 0.033

^1^ Age of coal miners without CWP in the year 1992 or at cessation of work in mine, age of coal miners with CWP at the time when they were compensated for the CWP; ^2^ smokers and ex-smokers, SD = standard deviation, iCWP = initial form of CWP, sCWP = simple CWP, PMF = progressive massive fibrosis, CWP & TBC = CWP with active tuberculosis; ^3^ *p*-value of statistical testing within the group of coal miners with CWP.

**Table 2:** The causes of death in the cohorts of coal miners with and without coal-workers´ pneumoconiosis (CWP).

Cohort	Coal Miners with CWP			Coal Miners without CWP		
Cause of Death According Diagnoses	Deaths	Age at Death (Years)	Exposure (Years)	Deaths	Age at Death (Years)	Exposure (Years)
(ICD-10)	N (%)	Mean ± SD	Mean ± SD	N (%)	Mean ± SD	Mean ± SD
Malignant neoplasms (C00–C97)	284 (32%)	66.7 ± 10.5	23.9 ± 9.2	437 (33%)	61.1 ± 7.4	26.0 ± 5.5
Diseases of the circulatory system (I00–I99)	332 (37%)	70.2 ± 11.4	24.7 ± 8.5	473 (36%)	60.9 ± 7.3	25.7 ± 5.5
Diseases of the respiratory system (J00–J99)	122 (14%)	70.5 ± 10.9	24.3 ± 9.3	77 (6%)	61.1 ± 8.5	26.8 ± 5.5
Diseases of the digestive system (K00–K93)	56 (6%)	59.0 ± 10.8	20.4 ± 8.4	126 (9%)	57.5 ± 7.8	24.7 ± 4.9
Injury, poisoning and certain other consequences of external causes (S00–T98)	51 (6%)	56.4 ± 14.1	18.4 ± 7.4	130 (10%)	53.4 ± 9.1	23.1 ± 6.1
Other	44 (5%)	67.0 ± 11.2	22.9 ± 8.9	77 (6%)	60.9 ± 7.8	25.1 ± 5.7
*p*-value (analysis of variance ANOVA)		<0.001	<0.001		<0.001	<0.001

ICD = International Classification of Diseases, CWP-coal-workers’ pneumoconiosis, N = numbers, SD = standard deviation.

**Table 3:** The comparison of total and specific mortality in black coal miners with and without the acknowledged coal-workers´ pneumoconiosis (CWP), and mortality of the general male population in the Czech Republic in the period 1992–2013.

Cohorts	Coal Miners with CWP			Coal Miners without CWP		
Cause of Death (ICD-10)	(N = 3476)			(N = 6687)		
	N_D_	SMR	95% CI	N_D_	SMR	95% CI
Total (A00–Y98)	889	1.10 **	1.02–1.17	1320	0.86 ***	0.82–0.91
Malignant neoplasms (C00–C97)	284	1.16 *	1.03–1.30	437	0.80 ***	0.73–0.88
Malignant neoplasm of lung (C33–C34)	116	1.70 ***	1.41–2.04	143	0.83 ***	0.70–0.98
Diseases of the circulatory system (I00–I99)	332	0.88 *	0.80–0.99	473	0.74 ***	0.77–0.92
Diseases of the respiratory system (J00–J99)	122	2.78 ***	2.32–3.31	77	1.08	0.85–1.35
Chronic obstructive pulmonary diseases (J44)	46	2.94 ***	2.15–3.92	22	0.82	0.51–1.23
Coal-workers´ pneumoconiosis (J60)	37	-	-	14	-	-
Diseases of the digestive system (K00–K93)	56	1.30	0.98–1.69	126	1.12	0.93–1.33
Age at study entry (years) (mean, SD) ^1^	58.9 (13.1)			47.3 (5.50)		
Age at death (years) (mean, SD)	67.4 (11.8)			59.9 (8.0)		
Exposure (years) (mean, SD) ^2^	23.6 (8.9)			25.5 (5.6)		
Proportion of smokers and ex-smokers (%) ^3^	68.9%			79.4%		

* *p* < 0.05; ** *p* < 0.01; *** *p* < 0.001, ^1^ Age of coal miners without CWP in the year 1992 or at cessation of work in mine, age of coal miners with CWP at the time when they were compensated for the CWP; ^2^ data on exposure found in 85% of deaths in the coal miners with CWP and 99.9% in the coal miners without CWP; ^3^ data on smoking found in 68% of deaths; ICD = International Classification of Diseases; SMR = standardized mortality ratio; SD = standard deviation; N = number of persons; N_D_ = number of deaths; iCWP = initial form of CWP; sCWP = simple CWP; PMF = progressive massive fibrosis; CWP & TBC = CWP with active tuberculosis.

**Table 4:** The comparison of total and specific mortality in black coal miners with the acknowledged coal-workers’ pneumoconiosis (CWP) and mortality of the general male population in the Czech Republic in the period 1992–2013 according to the severity of CWP.

Cause of Death (ICD-10)	The Categories of Coal-Workers‘ Pneumoconiosis (CWP)							
	iCWP		sCWP		PMF		CWP & TBC	
	(N = 606)		(N = 2465)		(N = 233)		(N = 172)	
	N_D_	SMR (95% CI)	N_D_	SMR (95% CI)	N_D_	SMR (95% CI)	N_D_	SMR (95% CI)
Total (A00–Y98)	63	0.85 (0.65–1.09)	653	1.01 (0.93–1.09)	96	1.22 (0.99–1.49)	77	2.00 *** (1.58–2.50)
Malignant neoplasms (C00–C97)	18	0.75 (0.44–1.18)	215	1.09 (0.95–1.25)	33	1.60 * (1.10–2.25)	18	1.70 * (1.01–2.68)
Malignant neoplasm of lung (C33–C34)	3	0.43 (0.09–1.26)	89	1.60 *** (1.29–1.98)	14	2.74 ** (1.50–4.59)	10	3.63 ** (1.74–6.66)
Diseases of the circulatory system (I00–I99)	17	0.68 (0.4–1.10)	259	0.86 * (0.76–0.97)	33	0.77 (0.53–1.08)	23	1.18 (0.75–1.77)
Diseases of the respiratory system (J00–J99)	5	1.48 (0.48–3.45)	78	2.22 *** (1.76–2.77)	22	4.62 *** (2.89–6.99)	17	8.02 *** (4.67–12.84)
Chronic obstructive pulmonary diseases (J44)	2	1.77 (0.21–6.39)	26	2.05 ** (1.34–3.00)	11	6.59 *** (3.29–11.80)	7	10.31 *** (4.14–21.21)
Coal-workers‘ pneumoconiosis (J60)	1	-	25	-	6	-	5	-
Diseases of the digestive system	10	1.55 (0.74–2.85)	39	1.18 (0.84–1.61)	2	0.72 (0.09–2.61)	5	2.68 (0.87–6.27)
Age—CWP ^1^	44.0 (9.0)		59.2 (12.4)		67.7(10.1)		57.9 (14.7)	
Age at death (years) (mean, SD)	54.6 (9.7)		68.1 (11.1)		73.4(9.7)		64.9 (13.9)	
Exposure (years) (mean, SD) ^2^	21.3 (8.1)		24.0 (8.9)		25.3 (8.9)		20.4 (8.6)	
Proportion of smokers/ex-smokers (%) ^3^	69.5%		71.0%		52.0%		74.1%	

* *p* < 0.05; ** *p* < 0.01; *** *p* < 0.001; ^1^ Age of coal miners with CWP at the time when they were compensated for the CWP; ^2^ data on exposure found in 85% of deaths; ^3^ data on smoking found in 68% of deaths; ICD = International Classification of Diseases; SMR = standardized mortality ratio; SD = standard deviation; N = number of persons; N_D_ = number of deaths; iCWP = initial form of CWP; sCWP = simple CWP; PMF = progressive massive fibrosis; CWP & TBC = CWP with active tuberculosis.
